# Temporal sylvian fissure arachnoid cyst in children: treatment outcome following microsurgical cyst fenestration with special emphasis on cyst reduction and subdural collection

**DOI:** 10.1007/s00381-022-05719-w

**Published:** 2022-11-09

**Authors:** Tadanori Tomita, Amanda M. Kwasnicki, Laura S. McGuire, Arthur J. Dipatri

**Affiliations:** 1 Division of Pediatric Neurosurgery, Ann & Robert H. Lurie Children’s Hospital of Chicago, 225 East Chicago Avenue, Chicago, IL 60611 USA; 2 Department of Neurological Surgery, Northwestern University Feinberg School of Medicine, Chicago, USA; 3 Department of Neurological Surgery, University of Illinois Chicago, Chicago, USA

**Keywords:** Arachnoid cyst, Children, Cyst fenestration, Micro-neurosurgery, Sylvian fissure, Subdural collection

## Abstract

**Objectives:**

Controversy remains regarding surgical managements of sylvian fissure arachnoid cyst (SFAC). This review presents our experience in the microsurgical fenestration of pediatric patients with SFAC to define surgical indication, and risks and benefits with special emphasis on postoperative subdural fluid collection (SDFC) and cyst size reduction.

**Methods:**

Thirty-four children with SFAC who underwent microsurgical cyst fenestration at a single institution over a 10-year period were retrospectively reviewed for their clinical presentation, neuroimaging findings, and postsurgical course. The SFACs were classified by a novel grading system based on the degree of arachnoid cyst extension from the sylvian fissure to the insular cistern shown on MR images: grade 0 — little or no prominence of sylvian fissure, grade I — SFAC confined to the sylvian fissure, grade II — SFAC partially extending to the insular cistern, grade III — SFAC extending to the entire insular cistern.

**Results:**

There were 26 males and 8 females. SFAC was present in the left side in 24. Twelve patients presented with cyst rupturing to the subdural space. Cyst grading did not show significant difference compared with rupture status (*p* > 0.9). All patients underwent microsurgical cyst fenestration. Postoperative SDFC is common but often resolved overtime in two-thirds of the cases with the mean average of 6 months. However, 3 patients had symptomatic postoperative SDFC and needed reoperation shortly after the first operation. Microsurgical cyst fenestrations for SFAC effectively resolved the presenting symptoms and often showed restorations of intracranial structures on follow-up imaging. Cyst resolution or reduction greater than 75% was noted in 61.8% of the patients postoperatively which was noted in a half of the SFAC of children even with age of 11 years or older. During the follow-up, no cyst recurrence or SDFC was noted. Patients with greater surgical reduction of cyst size tended to occur in younger children, and those with lower MR grade.

**Conclusion:**

Our results showed a high reduction rate of SFAC and brain re-expansion after microsurgical fenestration together with symptomatic improvements regardless the patient’s age. Considering the developing CNS during childhood, reductions of a large space-occupying lesion followed by restorations of the structural integrity of the developing brain are very desirable. However, a multi-center cooperative prospective longitudinal study on long-term comparative data of those treated and untreated of neuro-psychological outcome and cyst rupture incidence is needed.

## Introduction

Sylvian fissure arachnoid cyst (SFAC) is the most common type of intracranial arachnoid cyst (IAC), consisting of 47 to 66% of all pediatric IAC [1–3]. Presenting symptoms of SFAC vary from those of increased ICP to non-specific symptoms, such as psychomotor delay, behavioral disorder, and seizures. On the other hand, midline IACs, such as suprasellar, pineal, or posterior fossa location, commonly present with obstructive hydrocephalus or mass effects on local neural structures.

Much controversy remains regarding the management of SFAC, specifically the role of surgical interventions. In particular, when the child presents with a large space-occupying cyst in the middle cranial fossa with relatively insignificant symptoms, it may be challenging for clinicians to determine their recommendations. The reasoning of surgical intervention in such a case may be prophylaxis for future neurological deterioration or hemorrhagic rupture. However, there is no consensus in this regard, and its decision depends on the surgeon’s preference and previous experience, rather than on systematic protocols [4]. Incidental IAC is discovered by MR in 1.4% of adult population, and 34% of incidental IACs are located in the middle cranial fossa [5]. Also, there is a lack of clear-cut criteria to define the success of the surgery: improvement of clinical symptoms, cyst reduction, or the prevention of intracranial bleeding [6].

Following successful SFAC fenestration, a great majority of children’s preoperative headaches, emesis, decreased vision, cranial nerve palsy, and papilledema improve. On the other hand, the improvement of chronic symptoms, such as bone bulging, macrocephaly, developmental delay, and seizures, are much less [7, 8]. Cuny et al. conducted a prospective analysis of the cognitive and psychological profiles of 100 consecutive children. They reported that 50% of these children had neuropsychological difficulties and needed rehabilitation and/or psychotherapy for learning or behavior difficulties at initial assessment [9]. Of 34 of these 100 children, who underwent cyst fenestration, postoperative assessments showed improvement in 76% of surgically treated cohort regardless of the child’s age [10].

With regard to the natural history of arachnoid cysts, the stability of cyst size without intervention has been documented at 3–4 years follow-up [1, 2]. Asymptomatic patients with IAC, which were discovered incidentally, rarely increase in size over time. Al-Holou et al. reported only 11 (10%) of 110 IACs showed increase over time whereas 13 conversely decreased. Lee et al. reported even during infancy, 45% showed an interval increase among infants under than 12 months, but none showed enlargement among children older than 3 years of age [2].

Rupture, either spontaneous or post-traumatic, is a well-recognized complication of SFAC leading to subdural effusion or hematoma. Cress et al. reported that SFAC is the most common cause of subdural hygroma, or hematoma related to rupture of IAC, comprising 87% of cases, and the rupture is more common among children, with mean age 5.9 years during childhood [11]. Among all age groups, the rupture risk peaks at 10–19 years; however, rupture continues to occur throughout adult life, albeit with decreasing tendency with age [12]. Estimated rates of arachnoid cyst rupture were described at 2.3 to 6% in the literature [11, 13–15]. Al-Holou et al. reported only 1 case of post-traumatic hemorrhage among 309 patients retrospectively reviewed with a mean follow-up of 3.5 years [1]. At present, there are no prospective, extended longitudinal studies in the literature to accurately conclude the frequency of spontaneous rupture.

Cyst size reduction has been noted in most of the cases following cyst fenestration by either microsurgery or endoscopy [8]. The cyst size reduction is the reflection of the re-expansion of the chronically compressed brain. It remains unanswered if this reconstitution of the developing brain can alter the patients’ long-term psychomotor development.

Surgical intervention is accepted when patients present with evidence of increased ICP, such as headaches, papilledema, abducens palsy, or lethargy, or with acute presentation due to rupture of the cyst to the subdural space. On the other hand, for the cases presenting with incidental SFAC or ambiguous symptoms, surgical management remains controversial regardless of the size of SFAC and is often not recommended because of stable in size of SFAC over extended time and the potential surgical complications [1, 13].

Surgical procedures for SFAC include microsurgical or endoscopic cyst fenestration while cystoperitoneal (CP) shunts are not widely recommended because of shunt dependency. Cyst fenestration can be complicated by subdural effusion or hematoma, CSF leak from the surgical site, cranial nerve palsy, and failure of size reduction or recurrence of the cyst [1, 2, 16]. Following opening of the cyst membrane, which causes iatrogenic cyst rupture, subdural fluid collection (SDFC) naturally occurs. Development of these collections is thought to be due to the lack of immediate brain re-expansion and the chronic nature of the disease process. These postoperative SDFCs are often asymptomatic. However, with continuous CSF inflow to the subdural space, they may increase in size and become symptomatic, necessitating treatment. The frequency of symptomatic SDFC following SFAC fenestration ranges from 2 to 12.5% in the literature [3, 7 16–18]. However, asymptomatic postoperative SDFC is a very common finding on postoperative imaging, occurring in more than 80% of the cases [19].

This review of our surgical series of operative SFAC focuses on clinical outcome following microsurgical cyst fenestration, with particular emphasis on cyst size reduction, postoperative SDFC, and clinical outcome.

## Material and methods

A retrospective study was conducted of all patients who had microsurgical cyst fenestration of SFAC diagnosed and treated at a single neurosurgical center from 2010 through 2019. We included in this study only children who were treated with a temporal craniotomy and microsurgical cyst fenestration to the suprasellar cistern. Patients who underwent endoscopic cyst fenestration or primary CP shunt were excluded. Also, patients who underwent only outer membrane excision without fenestration were excluded. All patients underwent a keyhole temporal craniotomy under neuronavigational guidance for fenestrations by creating a cystocisternostomy. Presenting signs and symptoms, radiographic imaging studies, operative reports, and postoperative and ambulatory records were reviewed. Cyst location and size were reviewed by preoperative, postoperative, and subsequent follow-up magnetic resonance (MR) images. This investigation was approved by the Lurie Institutional Review Board (IRB #2020–3252).

### Imaging review

**Fig. 1:**
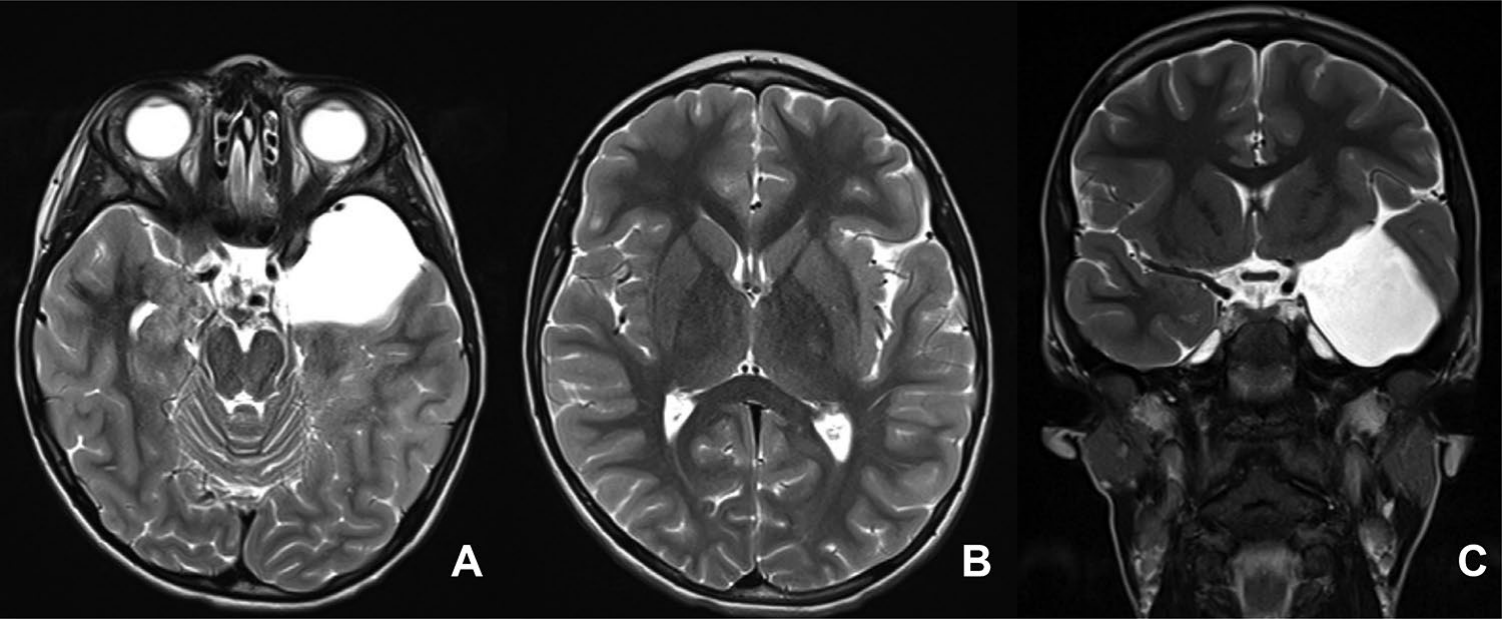
T2-weighted MR of group I sylvian fissure arachnoid cyst (SFAC) (axial images — **A** and **B**; coronal image — **C**). Note the cyst is localized at the proximal sylvian fissure. The insular fissure remains closed and the insular cortex is not exposed to the cyst

**Fig. 2:**
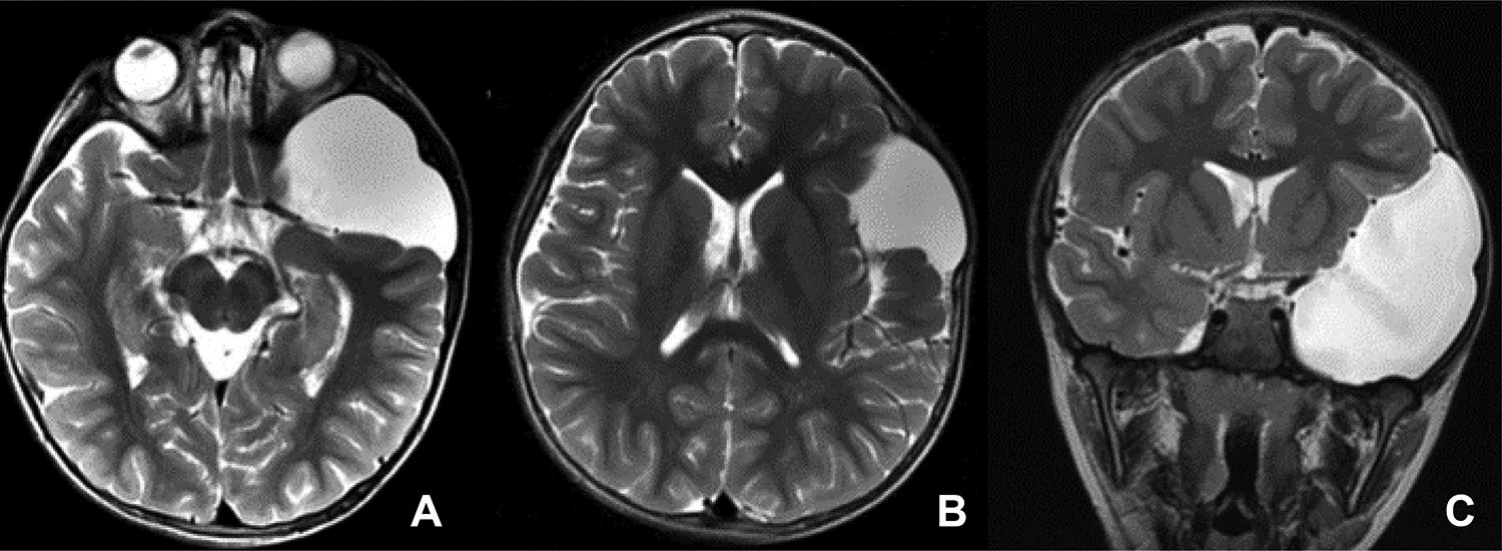
T2-weighted MR of group II sylvian fissure arachnoid cyst (SFAC) (axial images — **A** and **B**; coronal image — **C**). Note the cyst is extending to and opening the anterior insular fissure with displacement of the frontal and temporal opercula. The insular cortex is partly exposed to the cyst

**Fig. 3:**
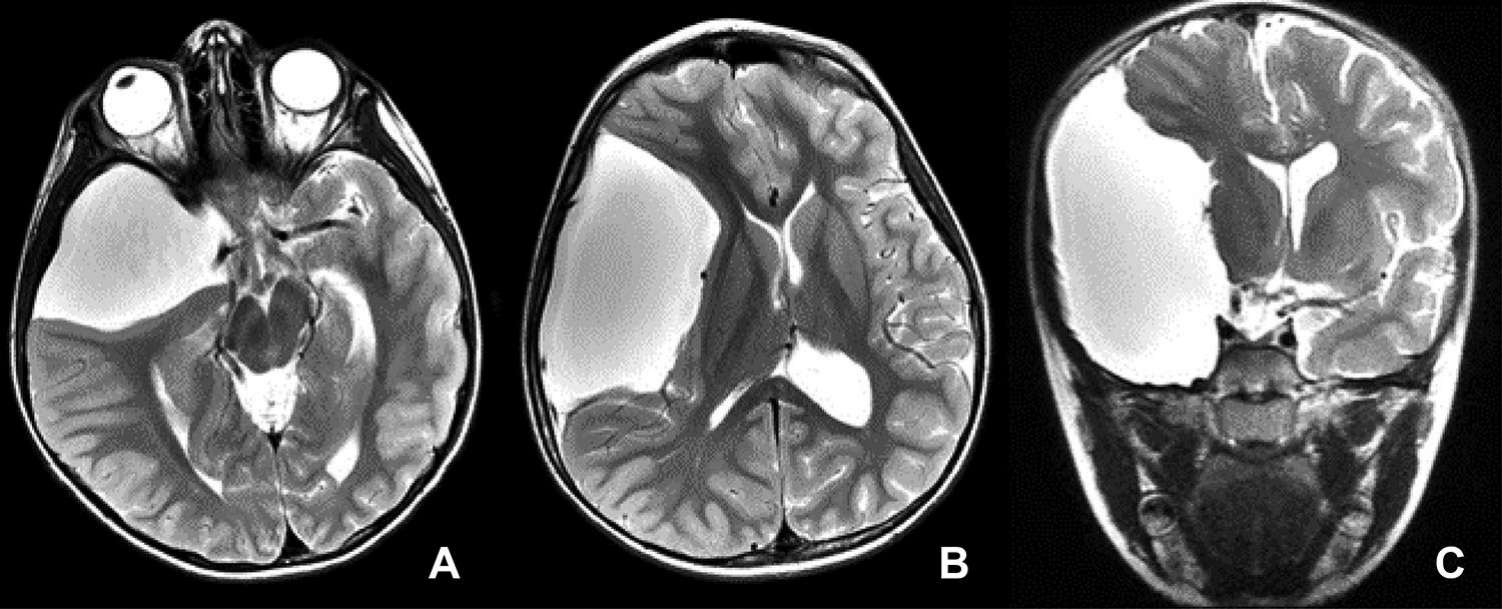
T2-weighted MR of group III sylvian fissure arachnoid cyst (axial images — **A** and **B**; coronal image — **C**). Note the cyst is extending to and opening the entire fissure by displacing the frontal, parietal, and temporal opercula. The insular cortex is wholly exposed to the cyst

Based upon the MR characteristics of arachnoid cyst extension from the sylvian fissure to the insular cistern, we developed our own classifications for SFACs into 4 grades as follows: Grade 0 — no or little enlargement of the sylvian fissure; grade I — cyst localized in the proximal sylvian fissure (Fig. 1); grade II — cyst extending from the sylvian fissure to the insular cistern with partial exposure of the insular cortex to the medial wall of the cyst (Fig. 2); grade III — the cyst from the sylvian fissure to the entire insular cistern exposing all or nearly the entire insular cortex to the medial cyst wall (Fig. 3).

Dependent on the presence and absence of SDFC over the cerebral convexity at presentation prior to craniotomy, SFACs were divided into “rupture” group and “non-rupture” group. Postoperative follow-up evaluations and MR images were done regularly, within 48 h after the surgery, 3 months, 6 months, 1 year, and subsequently annually for 5–6 years after the cyst fenestration. Cyst volume size was measured in the transverse, anteroposterior, and craniocaudal dimensions, at their largest diameter to compare preoperative measurements with subsequent ones. The final comparison of the changes of the cyst size was based on preoperative and the latest MR findings.

Demographic, clinical, and imaging characteristics were analyzed using summary statistics. Further univariate analysis to identify factors associated with cyst and subdural collection resolution were completed using chi-square analysis and independent samples *t*-test. Kaplan–Meier curve analysis was done to assess time to subdural collection resolution. Statistical analysis was performed using SPSS Statistics software (version 27, IBM Corporation, Armonk, NY).

## Results

### Patient demographics

There were thirty-four patients with SFAC who underwent a temporal craniotomy for microsurgical cyst fenestration. They were 26 (76.5%) male and 8 (23.5%) female. At the time of surgery, the average mean age was 9.4 years ± 4.8. Follow-up information was available from 4 months to 9 years (average 3.68 years) after the SFAC fenestration. Three patients had follow-up less than 6 months, while follow-up visits were a minimum of 1 year for the remainder.

Four patients (11.8%) were diagnosed incidentally with no or little symptoms referable to the cyst in spite of large cyst size and mass effect on the surrounding tissue. The remaining thirty patients presented symptomatically (88.2%): primary symptoms included 21 with headache ± nausea (61.8%), 3 with neurologic deficits (8.8%), 2 with cognitive dysfunction (5.9%), 4 with seizure or abnormal EEG (11.8%). Of those with neurologic deficit at presentation, one had abducens nerve palsy, another had ipsilateral visual disturbance, and the other presented with acute vision loss and stroke. Papilledema was documented in 6 patients, five of which occurred among the “rupture” group. Two patients were noted to have macrocephaly.

There were twelve (35.3%) patients that presented with a preoperative cyst rupture and SDFC over the cerebral convexity (“rupture” group). Within the “rupture” group, 4 (33.3%) had documented head trauma prior to presentation; a minor trauma in 3, and a football-related head concussion in one. Of the 22 patients with unruptured SFAC, 4 (18.2%) experienced minor trauma prior to presentation. The mean age was 8.4 years ± 4.8 among the “rupture” group and 7.9 years ± 5.3 among the “non-rupture” group without statistical difference (*p* = 0.384).

### Image findings

**Fig. 4:**
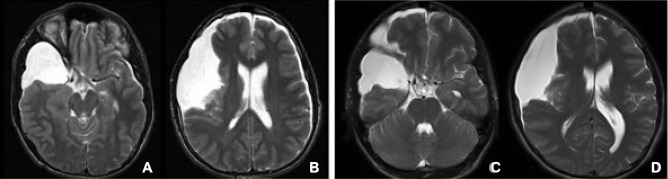
T2-weighted axial MR. A 17-year-old presented progressive headaches after mild head trauma 3 weeks prior. MR showed grade III SFAC of the right side with bilateral subdual collection (**A**). Five days later, MR showed resolution of left-sided subdural effusion with increased right-sided subdural effusion and midline shift

Twenty-four (70.6%) patients had left-sided arachnoid cysts; 10 (29.4%) were located on the right side. Preoperative MR showed grade I in 11 (32.4%), grade II in 9 (26.5%), and grade III in 14 (41.2%). There was no significant difference between cyst grading and rupture status (*p* = 0.598). The location of subdural collection was ipsilateral in 5 and bilateral in 7 at initial evaluation. But four of bilateral subdural collections became ipsilateral before the surgical intervention (Fig. 4).

**Fig. 5:**
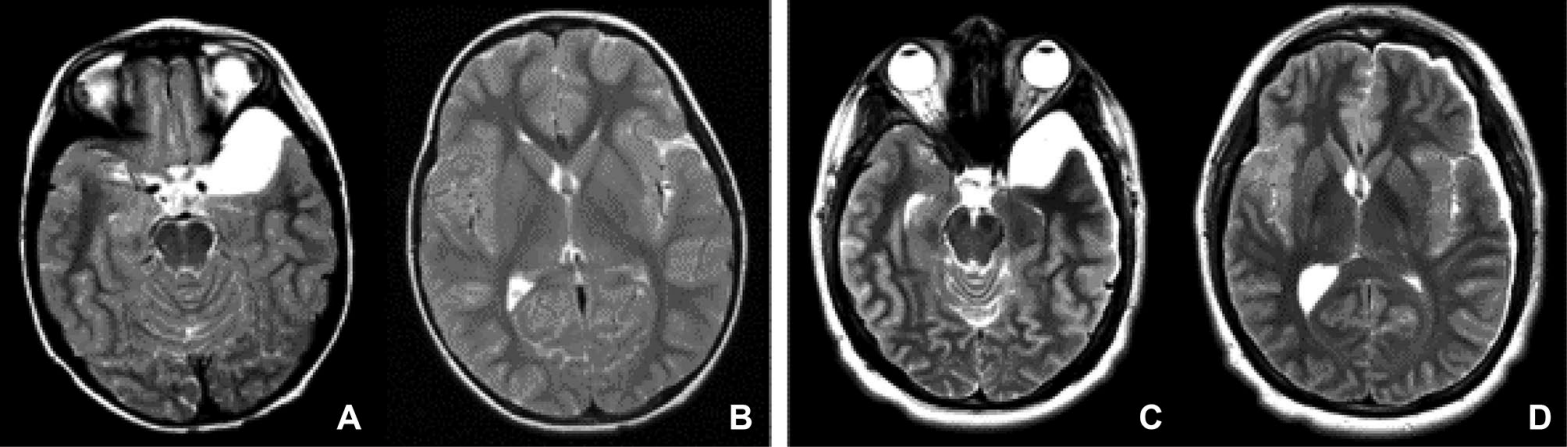
T2-weighted axial MR showing interval development of group I arachnoid cyst of the left side. He was found with incidental left SFAC at the age of 5 years when he had neck swelling (**A**). He did well until 7 years old; he developed headaches after diving to a pool which was persistent. MR then showed subdural collection and mild midline shift with a stable SFAC (**B**)

In 8 patients, previous MR images were available for review and had shown the presence of SFAC. They were obtained among five patients of the “non-rupture” group at 11 months, 2 years, 5 years, 6.5 years, and 10 years ago. Among them, interval increase of SFAC was noted in only one patient, a 10-year-old boy who had initial study done when he was 2 months of age. The remaining three patients of “rupture” groups had prior MR images obtained 10 to 12 years ago that showed no signs of cyst rupture on the initial images (Fig. 5).

### Surgical intervention

#### Pre-craniotomy surgical intervention

Three patients had urgent drainage before cyst fenestration due to acute symptomatic presentation. Two patients of the “rupture” group had an external subdural drainage (ESD) placed as an initial treatment. One of them experienced increased subdural effusion despite ESD for 3 days, and he subsequently underwent cyst fenestration. The other patient had 6 days of ESD, which was removed after symptomatic improvement; however, 2 months later, due to recurrent headaches and increasing subdural collection on a follow-up imaging, he underwent craniotomy for cyst fenestration.

**Fig. 6:**
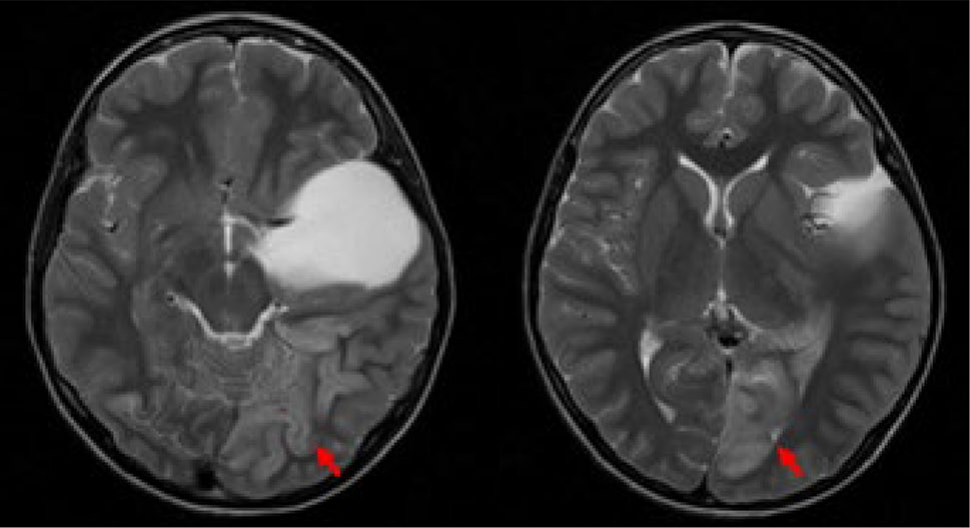
T2-weighted axial MR showing left-sided group II SFAC with left occipital lobe infarct (arrow)

The third patient, a 12-year-old male, had a non-ruptured SFAC and presented with acute headaches and homonymous hemianopia with evidence of ipsilateral posterior cerebral artery infarct on MR (Fig. 6). He underwent external cyst drainage (ECD). After 6 days with ECD, his headaches improved but his visual field deficit persisted. He subsequently underwent a microsurgical cyst fenestration and his visual field deficit resolved 3 months later.

#### Temporal craniotomy with microscopic cyst fenestration

A keyhole temporal craniotomy of size ranging from 2.5 to 3.0 cm in diameter was placed over the cyst dome using a neuronavigational system. Exposed dura uniformly showed bluish discoloration indicative of underlying fluid collection. The ICP was not measured, but it deemed elevated in all because the cyst wall bulged out upon the opening of the dura, or cyst fluid or subdural fluid gushed out under pressure. All non-rupture cysts contained crystal clear fluid. Among the “rupture group,” 10 patients showed clear xanthochromic subdural fluid while the remaining two had grossly hemorrhagic fluid but without clot formation. Only one patient showed the presence of an organized subdural membrane over the parietal cyst membrane.

**Fig. 7:**
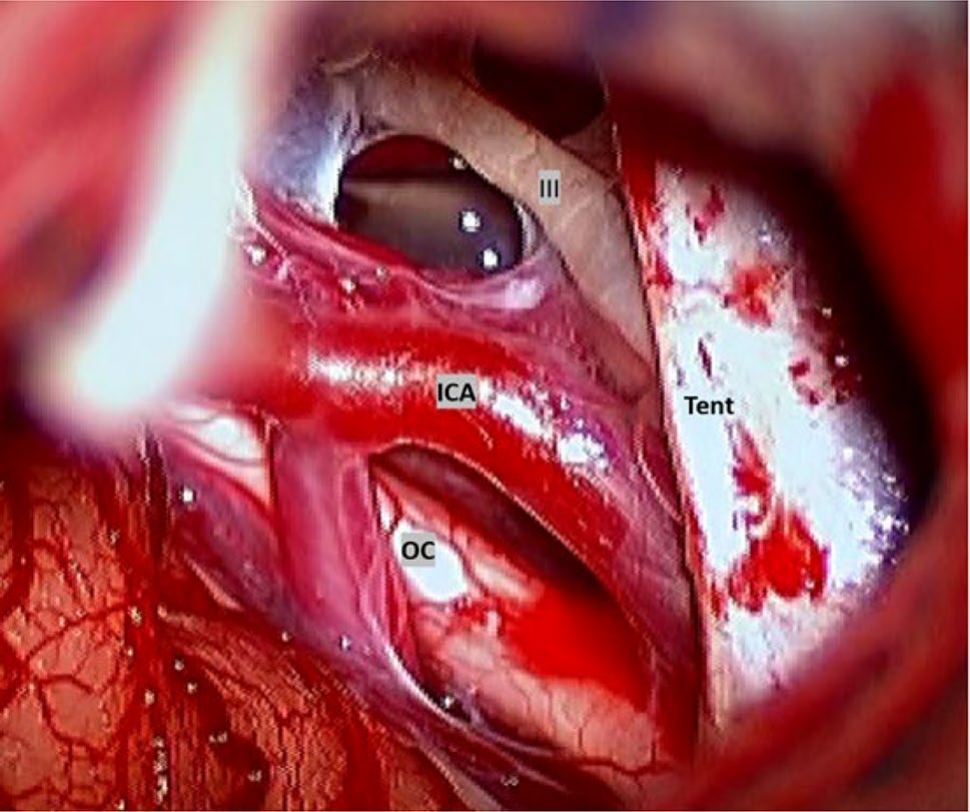
Intraoperative photograph after wide exposure of parasellar structures. Note the tentorial edge with the exposed internal carotid artery and its branches, the oculomotor nerve, and the lateral edge of the optic chiasm

For the cyst fenestration, the outer cyst membrane was partially excised, and the cyst space was entered. Then, the medial cyst wall next to the edge of the tentorium was approached under a surgical microscope. The medial cyst membrane adjacent to the suprasellar cistern at the tentorial edge was often thick, multilayered, and fibrous. It was sharply dissected and then partially excised to establish the maximum cystocisternostomy. Upon opening the medial cyst membrane, para-sellar structures, such as the ipsilateral carotid artery and its branches, the oculomotor nerve and the optic nerve, chiasm, and tract were exposed (Fig. 7). In some occasions, the surgical view showed the basilar artery posteriorly and the olfactory nerve anteriorly, and even the contralateral carotid artery, and the optic nerve. These neural and vascular structures were meticulously protected during cyst wall dissection.

#### Postoperative clinical course following cyst fenestration

Following the craniotomy, three patients (8.8%) had an unplanned return to the operating room due to postoperative symptomatic SDFC: two from the “non-rupture” group and one from the “rupture” group. One was a 4-year-old boy of the “non-rupture” group who developed acute headaches, lethargy, and bradycardia 3 days postoperatively due to an expanding ipsilateral hemorrhagic subdural effusion. He was treated with ESD for 7 days and his symptoms resolved. The ESD was removed and 2 years following the cyst fenestration, not only the subdural collection but the SFAC had resolved.

Another patient, a 7-year-old with a ruptured grade III arachnoid cyst, presented with headaches when MR showed a ruptured subdural collection over bilateral convexity. His symptoms improved and he was observed. However, 6 weeks later, he acutely developed severe headaches and MR showed increased midline shift with increased left convexity subdural collection and mild reduction of the left SFAC. He had a subdural membrane noted at craniotomy for fenestration. A cyst fenestration was done following the evacuation of the hemorrhagic subdural collection (no clot). Postoperatively, overnight he developed lethargy and left oculomotor nerve palsy, needing a re-craniotomy with ESD. He improved and hemorrhagic subdural fluid becomes clearer and the ESD was removed 14 days postop. SDFC resolved on 3-month postoperative imaging and his group III SFAC was reduced to group II.

The third patient was an 18-month-old girl, “non-rupture” group III SFAC, with macrocephaly and developmental delay, developed symptomatic bilateral subdural collection 4 weeks after the fenestration. She underwent burr hole and drainage of subdural collection with a placement of ESD. However, 2 weeks after removal of the ESD, she developed symptomatic SDFC, and had placement of a subdural peritoneal shunt. This was the only patient who was treated with shunting after cyst fenestration in our series.

Neurological complications included transient cranial nerve III palsy in 2 (5.9%) patients, which were transient and resolved in 2 months. Another patient had vertical diplopia, likely due to trochlear nerve manipulation, which resolved in 6 weeks.

Of 26 patients with preoperative headaches, 20 (77%) had resolved. Headaches improved and only intermittent or migraine-like headaches in 3 (2 grade I and 1 grade II), while headaches were persistent in 3 (2 grade I and 1 grade II). In all of grade III patients, preoperative headaches resolved. Three patients who presented with seizures showed no seizures during the 2.5 to 10 years follow-up, and only one remained on antiepileptic drugs. Macrocephaly stabilized in 2 patients. One patient who presented with cranial nerve six palsy preoperatively had resolution of the palsy postoperatively. However, all 3 patients with psychomotor delay/behavioral disturbance showed no or little improvement.

All 4 incidentally found SFAC remained asymptomatic, and their cyst size decreased by 90% or greater in 2 patients, 75% in 1 patient, and 50% in 1 patient.

### Outcome of postoperative subdural collection

Post-operatively, 30 patients developed cerebral convexity SDFCs (18 “non-rupture” group and 12 “rupture” group), while 4 patients, “non-rupture” group, showed little or no SDFC on postoperative imaging.

The SDFCs were present on immediate postoperative imaging while two patients developed a delayed SDFC after 7 and 10 days, respectively, both of which resolved without intervention. Six patients had persistent but decreased asymptomatic SDFCs, 4 of which were persistent for more than 5 years (2 patients grade I, 2 patient grade III).

**Fig. 8:**
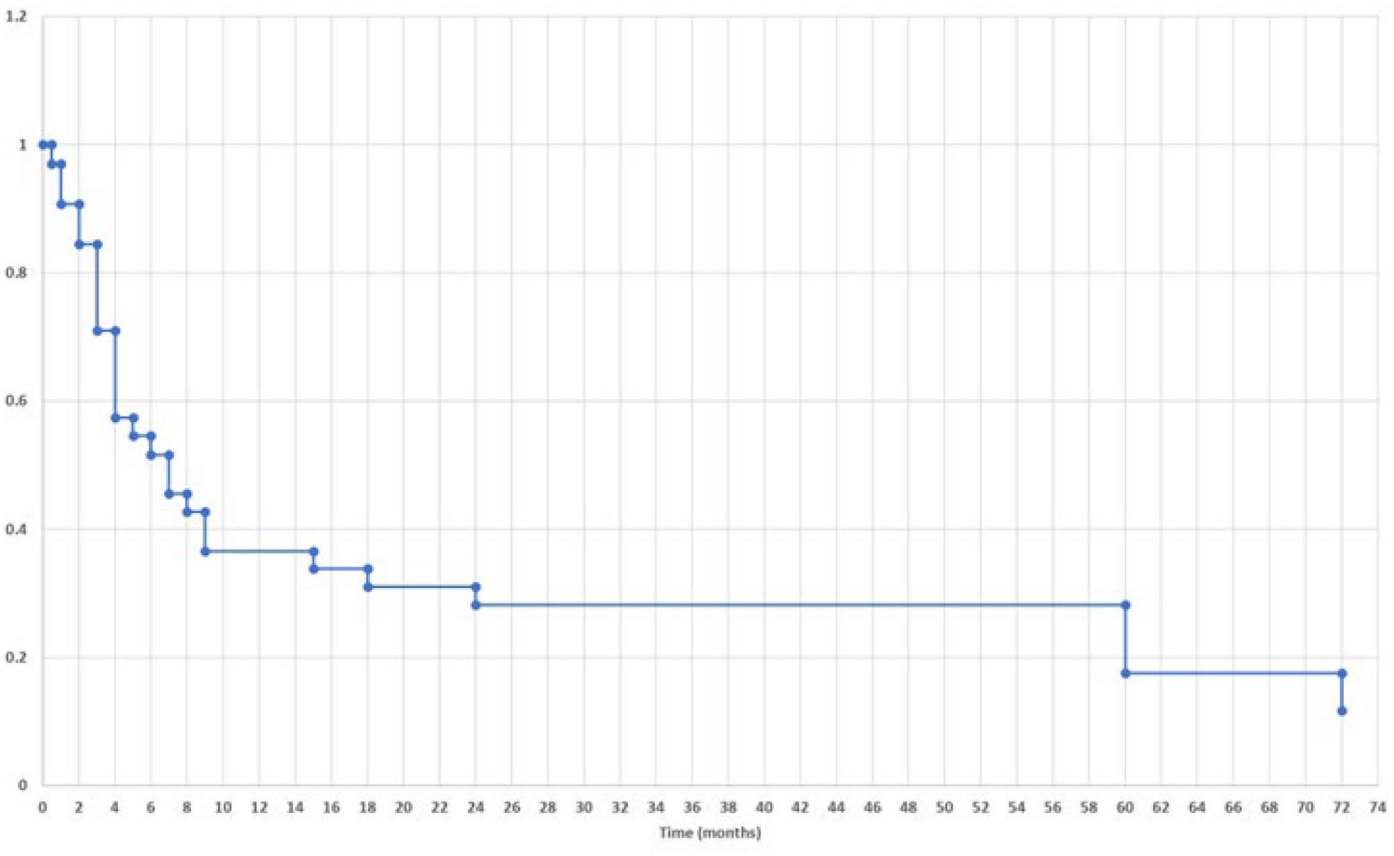
Time table for resolution of subdural fluid collection following SFAC fenestration

The remaining 24 (70.6%) of 30 SDFCs were resolved at various times during follow-up; 8 resolved in less than 3 months, 6 resolved between 4 and 6 months, and 5 resolved between 7 and 12 months; thus, 19 (79.2%) of the resolutions of subdural collection occurred within 1 year. The remainders, 2 were resolved between 13 and 24 months and 2 others were resolved 6 years after the cyst fenestration. Follow-up of a child with SDFC was too short. Figure 8 shows a life-table curve of the resolution of SDFC after cyst fenestration of SFAC. The median time for resolution of SDFC was 6 months.

### Outcome of postoperative cyst size

The changes in cyst volume were evaluated before and after the cyst fenestration. SFAC collapsed or resolved in 8 (23.5%), reduced more than 75% in 13 (38.2%), reduced to between 50 and 75% in 3 (8.8%), less than 50% reduction in 7 (20.6%), and remained unchanged in the 3 (8.8%). Thus, 21 (61.8%) patients showed quite significant cyst volume reduction greater than 75% of the preoperative size following microscopic cyst fenestration. The extent of the cyst reduction was compared with the MR grades, “rupture” vs. “non-rupture” status and the patient’s age, respectively (Table 1).

**Table 1:** Changes in cyst size volume before and after fenestration

**Cyst reduction**	**Grade**			**Rupture**		**Age (year) at surgery**		
	**Gr. I**	**Gr. II**	**Gr. III**	**No**	**Yes**	**1–5**	**6–10**	**11–17**
Collapse (8)	5	0	3	6	2	4	2	2
> 75% reduction (13)	4	5	4	11	2	2	5	6
50–75% (3)	0	1	2	1	2	0	1	2
< 50% (7)	2	1	4	3	4	2		5
No change (3)	0	2	1	1	2	1	1	1
*χ*2 test	*p* > 0.1			*p* < 0.01		*p* > 0.1		

A cyst collapse or a greater reduction more than 75% was noted in 9 of 11 grade I, 5 of 9 grade II, and 7 of 14 grade III. Although the lesser grade cysts appear to have greater cyst size reduction, the difference is not statistically significant (*p* > 0.1). Comparing the cyst rupture status at presentation, “non-rupture” groups had a significant cyst reduction (*p* < 0.01): 17 of 22 “non-rupture” group vs. 4 of 12 “rupture” group had the cyst collapse or a greater than 75% cyst reduction. The patient’s age was classified into 1–5 years (9 patients), 6–10 years (9 patients), and 11–17 years (16 patients), and compared with the cyst size outcome. Six (66.7%) of the 1–5 year group, 7 (77.8%) of the 6–10 year group, and 8 (50%) of 11–17 year old group had the cyst collapse or a greater than 75% reduction. Although the younger patients appear to have greater cyst size reduction, the difference is not statistically significant (*p* > 0.1).

## Discussion

### Classification of SFAC

SFACs originate at the sylvian fissure, which is bordered by the fronto-orbital cortex anteriorly and temporal lobe posteriorly. SFAC dorsally widens the sylvian fissure and further displaces the temporal, frontal, and parietal opercula, uncovering the insula cortex. Galassi et al. classified these cysts based on their size, location, and mass effect on the surrounding tissues and the degree of communication with the subarachnoid space [20]. Galassi’s type I cysts are small, located at the temporal tip, without evidence of mass effect. Galassi’s type II cysts are larger cysts, extending into the sylvian fissure, associated with minimal mass effect. Galassi’s type III cysts are even larger cysts that compress the adjacent frontal and parietal lobes and cause shift of the lateral ventricle across the midline. CT contrast cisternography and phase-contrast cine MR have shown communication between SFAC and the subarachnoid space [20, 21], though the exact communication sites remain unclear. Metrizamide CT cisternography demonstrated free communication between type 1 cysts and the subarachnoid space, while communication of Galassi’s type 2 and type 3 cysts with the subarachnoid space is delayed or absent, respectively.

The Galassi classification has been used widely but a new grading system may be required with advancements in neuroimaging. Amelot et al. used their interpretation of Galassi’s classification as follows: Galassi type I: small, spindle shaped, limited to the anterior middle cranial fossa. Galassi type II: superior extent along the sylvian fissure with displacement of the temporal lobe. C: Galassi type III: large, fills the entire middle cranial fossa, with displacement of not only the temporal lobe but also the frontal and parietal lobes [8]. The new SFAC classification used in this communication was based on the degree of cyst extension to the insular cistern. Multi-dimensional MR imaging was used to determine the size of the cyst, its location and extension from the sylvian fissure to the insular cistern, and exposure of the insular cortex. Grading increased with increase in cyst size. Ventricular effacement or midline shift is not noted in any of grade I SFAC and is very limited in degree with grade II SFAC while they occur invariably with grade III SFAC due to its magnitude.

### Arachnoid cyst etiology

There are various theories regarding enlargement of IAC, including fluid accumulation caused by osmotic pressure gradient, active fluid secretion by the cell lining [22, 23], and active CSF egress through persisting communication caused by venous or arterial pulsations with the one-way-valve mechanism between adjacent subarachnoid spaces and the cyst [24]. Communications between IAC and subarachnoid space were tested with CT cisternography and MR phase-contrast cine flow study, with jet-like flow voids in the cyst next to the basal cisterns demonstrating this communication [20, 25]. Suprasellar arachnoid cysts have also been shown with a slit-valve mechanism next to the basilar artery [26]. These theories, either cyst fluid production by cyst membrane or constant inflow of the CSF through the perivascular slit valve, however, fail to explain the lack of continuous enlargement of IACs. Unruptured IACs remain unchanged in size, while maintaining its expansile feature, year after year. We suspect there must be a two-way valve communication system. Halani et al. reported a case of a temporal arachnoid cyst with a small circular opening around a sylvian arterial branch that did not vary in size or position during the cardiac cycle [26]. Halani et al. speculate that this circular opening likely allowed bidirectional flow of CSF with equal resistance to flow in both directions, and the flow of CSF between the cyst and the surrounding sylvian cistern was probably a net of zero, keeping the arachnoid cyst stable and static in size [26].

### Surgical indication

Surgical indication for SFACs is multifactorial: patient’s age, cyst size, and symptomatology including headaches, focal neurologic deficit, seizures or developmental and cognitive deficits and cyst rupture.

Most SFACs present with relatively minor symptoms or identified incidentally on routine cranial imaging studies [1, 27, 28]. The literature indicate that it is rare for IAC to increase in size, particularly after the infantile stage [1, 2, 27]. During the follow-up period, IAC growth is unusual after found the age 3 or 4 years, and some may regress in size over time [1, 2]. On the other hand, young age patients, during the first 12 month in particular, are significantly associated with cyst enlargement [2]. SFACs are most conservatively treated and are least likely to undergo surgical intervention among IAC [29].

Much controversy remains for treatment of SFAC when they present with headaches because they are often subjective. However, headaches appear to be the best responder to surgical intervention. Multiple authors reported the resolution or improvement of headaches in 89–93% of patients following cyst fenestration [7, 18] which is similar to our results.

Defined criteria of symptomatic improvements of non-ICP-related symptoms have been absent [6]. Non-acute symptoms such as seizure, behavioral abnormality, and cognitive dysfunction respond to cyst fenestration to a lesser degree. According to Amelot et al., improvement was noted in 47% for patients with seizures, and 25% and 20% for those with behavioral disturbances and delay in cognitive development, respectively [8]. Similar lack of response of developmental delay was noted by others [7] and in our series. Seizure control after cyst fenestration showed mixed responses; Spacca et al. reported 7 of 8 patients had better seizure control or became seizure free [16] and all 3 in our series had seizure controlled. However, others reported less seizure control [7]. Cuny et al. reported neuropsychological testing before and after cyst fenestration showed improvement in 76% of 34 children [10]. They observed the rounded, convex shape of the cyst being more compressive and deleterious. Cyst size had very little neuropsychological impact while convex cysts were significantly associated with worse performance than nonconvex cysts. Cuny et al. emphasized the importance of neuropsychological testing at the time of decision-making for SFAC to determine surgical indication [9]. Among adult patients, based on long-term follow-up study of temporal arachnoid cyst 5 ± 2 years after temporal craniotomy and cyst fenestration, improved visual analogue scales for headaches and dizziness and quality of life were reported in the literature [30, 31].

Management of incidentally found large SFAC remains controversial. Preoperative ICP monitoring is not a common practice. Di Rocco et al. demonstrated constantly elevated ICP among larger SFACs like Galassi type 3 [32]. Large SFACs often unduly concern parents because of the potential risk for cyst rupture and/or inhibition of surrounding brain growth during the childhood. Four of the patients in our study with incidental, asymptomatic SFAC showed cyst resolution in 2 and a cyst size reduction greater than 75% in other 2. These patients remain asymptomatic.

Most acute symptoms such as severe headaches, papilledema, abducens palsy, and lethargy/irritability are due to ruptured cyst causing subdural effusion/hematoma and acute rise in the ICP. SFAC comprises the most common site of ruptured IAC [12]. The reported risk factors were large size and recent history of head trauma [11, 28]. However, they can rupture without recent history of head trauma [33]. Cress reported cysts greater than 5 cm were at a higher risk of rupture [11]. On the contrary, our data indicates that the “non-rupture” and “rupture” groups had similar distribution of the SFAC MR grading. Acute subdural hematoma and hygroma as a complication of IAC has been widely reported in the literature [14, 34–36]. Head trauma, even though minor, has been indicated to be a precipitating factor [12, 34, 37], although spontaneous rupture has also been described [35, 36]. Cyst ruptures occur more commonly in younger patients with its peak at 10–19 years old but can occur in adulthood [12]. Our data derived from only pediatric cohort showed there is no statistical difference in age distribution between “non-rupture” and “rupture” groups.

The incidence of long-term risks of ruptures has yet to be determined although the literature indicates risks of rupture are low. However, no data is available in the literature as to the true incidence of SFAC rupture based on extended longitudinal prospective studies. In general, “prophylactic” surgery for SFAC solely to prevent the future rupture has not been recommended in the literature. Although we did not observe delayed subdural hematoma or SDFC following microsurgical cyst fenestration in our series, cyst fenestration does not necessarily prevent subdural hematoma. Spacca et al. reported delayed postoperative traumatic subdural hemorrhage in four of 40 patients 3 to 60 months following an endoscopic fenestration even though 3 of them showed cyst disappearance or reduction after the initial fenestration [18]. Therefore, patients with SFAC are at risk of rupture throughout lifetime although its frequency has yet to be determined.

### Surgical therapy

Ali et al. reported no conspicuous difference in surgical outcome among surgical techniques for IAC (endoscopic, CP shunting, or craniotomy-based) [23, 38]. For the treatment of SFAC, there is no consensus regarding the optimal surgical approach among pediatric neurosurgeons [17]. Most contemporary authors, however, recommend cyst fenestration as the primary surgical intervention. Sufianov et al. reported a high rate of recurrence, 18.5%, following endoscopic cyst fenestration predominantly younger occurring in children under 3 years of age [39]. The same authors reviewed the literature and described reoperation rates associated ineffective endoscopic treatment being between 11.7 and 17% after the endoscopic procedure. Okano and Ogiwara reported the cysts decreased in size in all cases and disappeared in 11% following microsurgical fenestration. They reported none showed regrowth of the cyst [19].

However, Chen et al. compared endoscopic, microsurgery, and CP shunt by meta-analysis, and noted clinical improvement and cyst reduction were better after CP shunt. Also noted was that short-term complications were worse among the microsurgery group while long-term complications were worse after CP shunt [38]. In spite of excellent symptomatic improvement and cyst reduction rates [40], CP shunting has been avoided due to long-term concerns of shunt dependency, slit-cyst-like syndrome and development of Chiari I malformation [41, 42]. At times, the patients treated with CP shunt may require revision of the shunt or conversion to ventriculoperitoneal or lumboperitoneal shunt to obtain resolution of symptoms [42]. Only one patient in our study required CP shunting and has continued to clinically improve without complication.

Both endoscopic cyst fenestration and microsurgical cyst fenestration have been extensively described in the literature [8, 16, 29, 43–46]. Amelot et al. reported that the endoscopy group had earlier complications and a shorter event-free survival (EFS) time and with more subdural hematomas compared to the microsurgery group. The microsurgery group also showed a tendency for longer cystocisternostomy permeability than the endoscopy group [8]. Cuny et al., analyzing 34 children of SFAC treated with microsurgical fenestration (85%), endoscopic cyst fenestration (9%), or cystoperitoneal shunting (6%), described microsurgical fenestration is a safe and effective procedure [10]. On the other hand, others recommend the endoscopic approach as the best initial option, reserving microsurgical procedure as secondary options [7, 38]. A comparative study between microsurgical and endoscopic cyst fenestrations showed comparable results though open craniotomy provided better access for hemostasis [16].

Comparisons between microsurgical and endoscopic techniques are beyond the scope of this communication. We have used microsurgical cyst fenestration to establish a sufficient stoma between the cyst and basal cistern. Cyst wall resection without cyst fenestration tends to cause symptomatic SDFC [4, 32]. Cyst wall resection was limited to the parietal membrane but was not performed from the tentorial surface and the cortical surface to avoid hemorrhage. It is our opinion that the bimanual technique under a surgical microscope provided better control during SFAC fenestration because of often tough membranes and allowed safer widening of the fenestration. It is of paramount importance to avoid traumatizing neurovascular structures, and cranial nerves and optic pathway in the subarachnoid space. In this series, 2 patients had transient oculomotor nerve and trochlear nerve dysfunction, respectively. In spite of cautious manipulation and anatomical preservation of these structures, mechanical manipulation of the thick arachnoid membrane of the medial cyst wall may cause postoperative complications. Also, craniotomy provides a watertight dural closure better than endoscopic repair which may result in CSF leak. Other authors have found no differences in clinical outcomes among the various surgical techniques [29, 46].

### Surgical management of ruptured SFAC

Ruptured SFAC often present with severe symptoms. The recommended procedure of choice is burr hole/drainage or placement of a temporary ESD. Wu et al. stated that fenestration or resection of the arachnoid cyst membrane is not a requisite in patients with previous asymptomatic arachnoid cyst [12]. However, Cress et al. reported a high failure rate after burr hole/drain, in 4 out of 5, needing subsequent craniotomy [11]. Parch et al. reported excellent or good results following subdural fluid evacuation alone in 13 cases and no surgical treatment in 2 cases. Among them, only one patient underwent additional fenestration of the cyst wall [15]. On the other hand, Balestrino et al. stated microsurgical fenestration as a treatment of choice for ruptured SFAC to control the brain compression and lower the risk of recurrent rupture [33]. The same authors reported 9 cases of ruptured SFAC who were conservatively treated and attained subsequent regression of subdural collection with cyst size either stable or reduced [33]. Similarly, Maher et al. reported spontaneous resolution of subdural effusion without surgery. They stated an initial decision to manage without surgery is likely to result in a good outcome with symptom resolution in many cases [47].

### Post-fenestration subdural effusion reduction

Creation or expansion of subdural space is naturally common after decompression of a large space-occupying lesion such as SFAC, resulting in iatrogenic subdural effusion, which is one of the reasons against surgical treatment of asymptomatic SFAC. Tamburrini et al. [4] attributed the development of postoperative subdural hygroma to the opening of the external membrane of the IAC predisposing CSF fluid to accumulate within the subdural space where its absorption is insufficient [44]. Post-fenestration SDFC is a very common finding on postoperative imaging. Okano and Ogiwara reported that SDFC was identified in 82.1% at the immediate postoperative period and was asymptomatic in all cases but one [19]. They reported these SDFC disappeared in 83% and decreased in size in 13% in the long-term follow-up which is similar as our results [19]. In our cohort, all but 4 “non-rupture” SFAC showed resolution of the subdural effusion over various times with mean of 6 months. Only 4 patients of the “non-rupture” group showed persistent SDFC up to 7 years follow-up, which was comparable with the “rupture” group. None of the patients with persistent subdural effusion developed any neurological symptoms.

### Post-fenestration cyst reduction

The literature indicates various rates of the cyst reduction after cyst fenestration of SFAC. Spacca et al. reported SFAC disappeared in 5 patients, was reduced in 24, was unchanged in 9, and increased in 2 among 40 patients who underwent an endoscopic fenestration [18]. Schultz et al. reported following predominantly by endoscopic cyst fenestration, resolution of more than 50% reduction in 16 (39%) of 41 children with SFAC while 25 showed less than 10% reduction of the cyst [7]. Balestrini et al. reported cyst volume reduction in Galassi grade in 37% following microsurgical fenestration [33]. Cuny et al. reported cyst volume reduction 50% or greater among 59% of children following predominantly microsurgical fenestration [10].

Our current study showed several factors influence the post-fenestration cyst reduction. They are SFAC grades, “rupture” vs. “non-rupture,” and patient’s age. The cohort of lower grade showed more cyst size reduction postoperatively; cyst disappearance or more than 75% reduction were noted in 81.8% among grade I, in 55.6% among grade II, and in 50% among grade III. Also, we noted a younger age tends to show better cyst reduction, albeit no statistical significance (*p* > 0.2); dividing the age 10 years or younger vs. older, greater than 75% reduction was noted in 72.2% and 50%, respectively. The reduction of the cyst is significantly greater among the “non-rupture” group; a greater than 75% of the cyst reduction was noted in 72.7% of the “non-rupture” group while only 33.3% among the “rupture” group (*p* < 0.01).

Several authors have compared postoperative cyst volume with clinical improvement. In one study, even a cyst size reduction of greater than 10% was associated with significant improvement in symptomatic patients [7, 48], especially in those with symptoms related to increased ICP, acute neurologic deficit, and macrocrania. Less improvement was noted in those with temporal lobe epilepsy, behavioral abnormalities, and psychomotor retardation [48]. Others have demonstrated an association between clinical improvement and over 50% cyst volume reduction [49]. Cyst volume reduction can be detected immediately following operative intervention, however, may continue to reduce in size during the later follow-up period [45].

Cuny et al. reported that correlation analyses on the entire cohort of childhood SFAC revealed that a higher reduction of cyst volume was significantly associated with a larger Galassi type, an improvement in verbal episodic memory, and a reduction in delinquent behavior [10]. In contrast, other authors identified no correlation in decrease in cyst volume and clinical improvement when evaluated by psychological tests [50].

### Limitation of the current study

This is a retrospective review of the surgical experience at a single center. Each case is well known by the authors in regard to clinical presentation and outcome. There are several limitations to this study. This study only reviewed the characteristics of those patients who underwent microsurgical cyst fenestration. Patients with SFAC without surgery and those treated with primary CP shunting or neuroendoscopic cyst fenestration at the same time period were not included in this study. Further, we had no data of psychomotor developmental assessments of each child before and after cyst fenestration. Because there is no consensus in the literature and no standard protocol regarding surgical intervention, the surgical decision was made in this series based on the discretion of the attending surgeons (TT, AJD) based on the cyst size, and presenting symptoms of the patient. Although our patients did not experience cyst recurrence and SDFC in long-term follow-up, the follow-up period may be too short to conclude the efficacy of the treatment provided.

## Conclusion

Controversy remains regarding the management and treatment of SFACs, specifically regarding the role of surgical intervention for patients with relatively minor presentation. Although it remains controversial, several authors recommend cyst fenestration for asymptomatic SFAC because of improved cyst size reduction and low risk of complications [7, 32] and improved neuropsychological disorders [10]. The complication rates presented in this report were low and cranial nerve deficits (3rd and 4th nerve) were transient and resolved spontaneously. Because of watertight closure of the dura, we had no cases of CSF leak or pseudomeningocele from surgical sites as noted by others [16]. Symptomatic improvements and reductions of the cyst were noted in a great majority of our patients. SDFC postoperatively is common but often resolved overtime in a great majority of cases. Although 3 patients in this series required a second surgery for postoperative SDFC, microsurgical cyst fenestration for SFAC is effective to resolve the presenting symptoms. Considering the long future of children, it is ideal to provide the optimal environment for brain developments, particularly for children with large SFAC of grades II and III by reducing the space-occupying lesion and reconstituting the normal brain morphology. Reliable data for cyst rupture rates and psychomotor developmental assessments is not available, and a multi-center cooperative longitudinal prospective study is needed for children with SFACs.

## Data Availability

Available upon request.

## Abbreviations

CP: Cystoperitoneal
CSF: Cerebrospinal fluid
ECD: External cyst drainage
ESD: External subdural drainage
IAC: Intracranial arachnoid cyst
SFAC: Sylvian fissure arachnoid cyst
SDFC: Subdural fluid collection
